# *Phlomis fruticosa * L. exerts *in vitro* antineurodegenerative and antioxidant activities and induces prooxidant effect in glioblastoma cell line

**DOI:** 10.17179/excli2021-4487

**Published:** 2022-02-14

**Authors:** Dejan Stojkovic, Danijela Drakulic, Maria Inês Dias, Gokhan Zengin, Lillian Barros, Marija Ivanov, Uroš Gašic, Nemanja Rajcevic, Milena Stevanovic, Isabel C.F.R. Ferreira, Marina Sokovic

**Affiliations:** 1 Department of Plant Physiology, Institute for Biological Research “Siniša Stankovic” - National Institute of Republic of Serbia, University of Belgrade, Bulevar despota Stefana 142, 11000 Belgrade, Serbia; 2 Institute of Molecular Genetics and Genetic Engineering, University of Belgrade, Vojvode Stepe 444a, 11042 Belgrade 152, Serbia; 3 Centro de Investigação de Montanha (CIMO), Instituto Politécnico de Bragança, Campus de Santa Apolónia, 5300-253 Bragança, Portugal; 4 Physiology and Biochemistry Research Laboratory, Department of Biology, Science Faculty, Selcuk University, Campus, 42130, Konya, Turkey; 5 University of Belgrade, Faculty of Biology, Studentski trg 16, 11158 Belgrade, Serbia; 6 Serbian Academy of Sciences and Arts, Kneza Mihaila 35, 11000 Belgrade, Serbia

**Keywords:** Phlomis fruticosa, antioxidant, antineurodegenerative, glioblastoma, prooxidant, phenolic compounds

## Abstract

Despite the significant advances in drug development we are witnessing the inability of health systems to combat both neurodegenerative diseases and cancers, especially glioblastoma. Hence, natural products are comprehensively studied in order to provide novel therapeutic options. This study aimed to explore anti-neurodegenerative and anti-glioblastoma potential of extract of *Phlomis fruticosa *L. using *in vitro* model systems. It was found that the methanol extract of *P. fruticosa* was able to efficiently reduce activities of enzymes linked to neurodegenerative disease including acetylcholinesterase, butyrylcholinesterase and tyrosinase. Furthermore, *P. fruticosa* extract has shown excellent antioxidant potential, as evidenced by six different methods. Analysis of cytotoxic effect of *P. fruticosa* extract on A172 glioblastoma cell line revealed that the concentration of the extract necessary for 50 % inhibition of A172 growth (IC_50_) was 710 μg/mL. The extract did not induce changes in proliferation and morphology of A172 glioblastoma cells. On the other side, production of ROS was increased in A172 cells treated with the extract. Observed cytotoxic effect of *P. fruticosa* extract might be based on increase in ROS generation upon treatment. Quantitative chemical analysis revealed the presence of twelve different polyphenols with the *cis* 3-*O*-caffeoylquinic acid being the most abundant. This study provided scientific evidence for further exploration of *P. fruticosa* as a promising natural anti-neurodegenerative therapeutic option.

## Introduction

Due to the uprising number of elderly population worldwide we are witnessing an increase in the number of neurodegenerative disease patients including those suffering from Alzheimer (AD) and Parkinson diseases (van Bulck et al., 2019[37]). Oxidative stress, marked by increased oxidative damage to lipids, proteins and DNA, is one of the factors implicated in the pathogenesis of neurodegenerative diseases. Proteins amyloid beta and α-synuclein are key factors in Alzheimer and Parkinson diseases, respectively, and they are proved to induce generation of reactive oxygen species (ROS) by interacting with redox-active metal ions (Barnham et al., 2004[2]). Recent reviews highlighted the potential of different natural products to be used as part of neurodegenerative disease therapy (Li et al., 2019[15]; Stojković et al., 2020[31]), some of them, led by polyphenols, due to their strong antioxidant properties (Devi et al., 2021[8]; Matsuzaki and Ohizumi, 2021[19]). Current therapeutic strategy for AD includes acetylcholinesterase inhibitors such as donepezil, rivastigmine, and galantamine, drugs able to delay the disease progression by treating the symptoms not the disease (Moss, 2020[20]). At the same time, inhibitors of butyrylcholinesterase enzyme, which hydrolyzes acetylcholine in AD, are thoroughly being studied as indispensable parts of therapeutic strategies in AD (Li et al., 2017[16]). This strategy is also known as cholinergic hypothesis. Tyrosinase is a key enzyme in the melanin synthesis pathway, with interference in melanin biosynthesis recorded in neurodegenerative diseases (Bonesi et al., 2018[5]), with natural products once again seen as an indispensable source of enzyme inhibitors and potential therapeutics (Zengin et al., 2019[39]).

The transcriptomic meta-analyses pointed that the significant number of genes were deregulated in the same direction in both AD and glioblastoma (GBM) and epidemiological studies indicated that AD patients have higher risk of developing GBM (Sánchez-Valle et al., 2017[25]). Besides being the most common brain tumor, GBM is one of the deadliest human cancers with the poorest average survival - approximately 14-15 months from the diagnosis (Vilchez et al., 2021[38]), and with 5 year survival incidence lower than 7 % (Ostrom et al., 2020[22]). The current GBM therapeutic strategy covers surgical resection, concurrent radiotherapy and chemotherapy with temozolomide. However, if tumors progress despite the first-line therapy, therapeutic strategies are quite limited and exhausted (Birzu et al., 2020[4]). It has been revealed that dietary polyphenols exert antitumor activity due to cytotoxic mechanism based on ROS induction (NavaneethaKrishnan et al., 2019[21]). This suggests that natural products, especially polyphenol-rich, should be intensely studied as a possible therapeutic alternative in treatment of GBM that has been previously addressed by Abbas et al. (2020[1]) and Stojkovic et al. (2021[32]).

Genus *Phlomis* L. (*Lamiaceae*) comprises a range of species used worldwide in traditional medicines due to their health beneficial properties as reviewed previously by Limem-Ben Amor et al. (2009[17]). *Phlomis fruticosa* L. is medicinally used due to its anti-tussive properties while its leaves are eaten as a paste sauce ingredient in Italy. Numerous bioactive compounds have been previously detected in this species including polyphenols: chryseriol and its glycosides, hesperetin and naringenin (Limem-Ben Amor et al., 2009[17]). 

This study aimed to explore the *Phlomis fruticosa* L. methanolic extract as a possible antioxidant, antineurodegenerative and anti-glioblastoma agent and to reveal its phenolic constituents.

## Material and Methods

### Collection and extraction of the plant material

*Phlomis fruticosa* L. (*Lamiaceae*) aerial parts were collected during the flowering period, near Bar (Montenegro) in 1998, as previously published (Ristic et al., 2000[24]). The extract was prepared as described previously (Stojković et al., 2021[34]). 

### Biological activities evaluation

An antioxidant (DPPH and ABTS radical scavenging, reducing power (CUPRAC and FRAP), phosphomolybdenum and metal chelating (ferrozine method)) and enzyme inhibitory activities (cholinesterase (Elmann's method), tyrosinase (dopachrome method)) were determined using the methods previously described by Uysal et al. (2017[36]).

### DPPH (1,1-diphenyl-2-picrylhydrazyl) radical scavenging assay

The sample solution was added to 4 mL of 0.004 % methanol solution of DPPH. The sample absorbance was read at 517 nm after 30 min incubation at room temperature (RT) in the dark. DPPH radical scavenging activity was expressed as milligrams of trolox equivalents per gram of the extract (mg TE/g extract).

### ABTS (2,2′-azino-bis(3-ethylbenzothiazoline) 6-sulfonic acid) radical scavenging assay

Briefly, ABTS+ was produced by reacting 7 mM ABTS solution with 2.45 mM potassium persulfate and the mixture was allowed to stand for 12-16 minutes in the dark at RT. Prior to beginning the assay, ABTS solution was diluted with methanol to an absorbance of 0.700 ± 0.02 at 734 nm. Sample solution was added to ABTS solution (2 mL) and mixed. The sample absorbance was read at 734 nm after 30 min incubation at RT. The ABTS radical scavenging activity was expressed as milligrams of trolox equivalents per gram of the extract (mg TE/g extract).

### CUPRAC (cupric ion reducing activity) activity assay

For CUPRAC assay sample solution was added to the reaction mixture containing 10 mM CuCl_2_ (1 mL), 7.5 mM neocuproine (1 mL) and 1 M NH_4_Ac buffer pH 7.0 (1 mL). The blank was prepared by adding sample solution (0.5 mL) to the premixed reaction mixture (3 mL) that did not contain CuCl_2_. After 30 min of incubation at RT the sample and blank absorbances were read at 450 nm and the absorbance of the blank was subtracted from that of the sample. CUPRAC activity was expressed as milligrams of trolox equivalents per gram of the extract (mg TE/g extract).

### FRAP (ferric reducing antioxidant power) activity assay

The sample solution was added to FRAP reagent (2 mL) containing 0.3 M acetate buffer pH 3.6 and 10 mM 2,4,6-tris(2-pyridyl)-S-triazine (TPTZ) in 40 mM HCl and 20 mM ferric chloride in the ratio of 10:1:1 (v/v/v). Then, the sample absorbance was read at 593 nm after 30 min incubation at RT. FRAP activity was expressed as milligrams of trolox equivalents per gram of the extract (mg TE/g extract).

### Phosphomolybdenum method

The sample solution was combined with 3 mL of the reagent solution (0.6 M sulfuric acid, 28 mM sodium phosphate and 4 mM ammonium molybdate). After 90 min of incubation at 95 °C the sample absorbance was read at 695 nm. The total antioxidant capacity was expressed as millimoles of trolox equivalents per gram of the extract (mmol TE/g extract).

### Metal chelating activity assay

Briefly, the sample solution was added to 0.05 mL of 2 mM FeCl_2_ solution and the reaction was initiated by addition of 0.2 mL of 5 mM ferrozine. Blank was prepared by adding 2 mL of the sample solution to 0.05 mL of 2 mM FeCl_2_ solution and 0.2 mL water without ferrozine. Then, the sample and blank absorbances were read at 562 nm after 10 min incubation at RT. The absorbance of the blank was subtracted from that of the sample. The metal chelating activity was expressed as milligrams of EDTA (disodium edetate) equivalents per gram of the extract (mg EDTAE/g extract).

### Cholinesterase (ChE) inhibitory activity assay

The sample solution was mixed in a 96-well microplate with 125 µL DTNB (5,5-dithio-bis(2-nitrobenzoic) acid (Sigma, St. Louis, MO, USA) and 25 μL AChE (acetylcholinesterase (Electric ell acetylcholinesterase, Type-VI-S, EC 3.1.1.7, Sigma)) or 25 μL BChE (butyrylcholinesterase (horse serum butyrylcholinesterase, EC 3.1.1.8, Sigma)) solution in Tris-HCl buffer pH 8.0 and incubated for 15 min at 25 °C. With the addition of 25 μL acetylthiocholine iodide (ATCI, Sigma) or 25 μL butyrylthiocholine chloride (BTCl, Sigma) the reaction was initiated. Blank was prepared by adding the sample solution to reaction reagents without adding the enzyme solution (AChE or BChE). The sample and blank absorbances were read at 405 nm after 10 min incubation at 25 °C. The absorbance of the blank was subtracted from that of the sample. The cholinesterase inhibitory activity was expressed as galanthamine equivalents per gram of the extract (mgGALAE/g extract).

### Tyrosinase inhibitory activity assay 

The sample solution was mixed in a 96-well microplate with 40 μL tyrosinase solution (Sigma) and 100 μL phosphate buffer pH 6.8 and incubated for 15 min at 25 °C. With the addition of 40 μL L-DOPA (Sigma) the reaction was initiated. Blank was prepared by adding the sample solution to the reaction reagents without adding the enzyme solution (tyrosinase). After 10 min incubation at 25 °C the sample and blank absorbances were read at 492 nm. The absorbance of the blank was subtracted from that of the sample. The tyrosinase inhibitory activity was expressed as kojic acid equivalents per gram of the extract (mgKAE/g extract).

### DCF assay for oxidative stress

Dichlorofluorescein (DCF) assay was performed as described by Popovic et al. (2019[23]) with some modifications. Briefly, 8x10^3^ A172 cells were plated per well in dark 96-well plate. After 24 h, the cells were washed with 1×PBS and incubated with 50 µM DCFH-DA in PBS at 37 °C for 30 min. Then, the cells were washed with 1×PBS and fresh medium alone or fresh medium with DMSO (vehicle control), the extract (IC_50_ concentration) or 1 mM H_2_O_2_ were added on the cells. Immediately after administration, fluorescence was measured on the microplate reader Infinite 200 PRO. Readings were taken every 5 min for 180 min with excitation at 485 nm and emission at 530 nm. The area under the curve (AUC) was determined using following formula: AUC = [R1/2+sum (R2:Rn−1)+ Rn/2]×CT; R1 - the fluorescence reading at the initiation of the reaction, Rn - the fluorescence reading at the end of the measurement, CT - cycle time in minutes. The AUCs for the extract and 1 mM H_2_O_2_ were presented as fold change of the AUC for treatment with DMSO (arbitrarily set at 1). Treatments with medium only, DMSO and 1 mM H_2_O_2_ were done in 4 replicates and treatment with the extract was done in 8 replicates. Results are presented as the mean ± SD of four independent experiments, *p < 0.05. 

### Investigation of the cytotoxic effect of P. fruticosa extract

Cytotoxic effect of *P. fruticosa* extract on A172 glioblastoma cell line was analyzed by Crystal violet assay. The cells were grown in high-glucose Dulbecco's Modified Eagle Medium (DMEM) supplemented with 10 % fetal bovine serum (FBS), 2 mM L-glutamine and 1 % penicillin and streptomycin (Invitrogen) at 37 °C in 10 % CO_2_. In a 96-well plate 4 × 10^3^ cells were seeded per well. After 24 hours, fresh medium with different concentrations of the extract (500-1000 μg/ml) dissolved in dimethyl sulfoxide (DMSO) was added to the cells. After 48h of incubation of the cells with the extract, cells were washed twice with phosphate buffered saline (PBS) and stained for 15 min at RT with 0.5 % crystal violet staining solution. After removal of crystal violet the cells were washed in a stream of tap water and air-dried at RT. The absorbance of dye dissolved in methanol was measured at 590 nm in a microplate reader Infinite 200 PRO. Control A172 cells contained the same percentage of DMSO in medium as treatment with the highest concentration of the extract and the concentration of DMSO in the assay did not exceed 0.5 %. Four independent experiments were performed and the experiments were done in triplicate for each concentration of the extract. The results were expressed as IC_50_ value in μg/mL.

### Immunocytochemistry

In a 12-well plate 2.5×10^4^ A172 cells were seeded per well on coverslips. After 24 h, the cells were treated with the IC_50_ concentration of the extract or vehicle DMSO. After 48 h, the cells were fixed in 4 % paraformaldehyde for 15 min at RT and washed 3 times for 20 minutes in 1xPBS. Cells were permeabilized 10 minutes in 0.2 % Triton X-100 in PBS and blocked at RT for 1 h in 10 % normal goat serum/1 % bovine serum albumin (BSA) in PBS. Rabbit anti-Ki67 antibody (Abcam) and mouse anti-tubulin antibody (Abcam) were diluted 1:250 and 1:100, respectively, in PBS containing 1 % BSA/0.1 % Triton X-100 and applied on the cells for 1h at RT (anti-Ki67 antibody) or overnight at 4 °C (mouse anti-tubulin antibody). After incubation with anti-Ki67 antibody, the cells were washed 3 times for 15 minutes with 0.1 % Triton X-100 in PBS and afterwards incubated for 1 h with anti-rabbit secondary antibody conjugated with Alexa FluorH 488 (Invitrogen, diluted 1:500 in 1 % BSA/0.1 % Triton X-100 in PBS). Later, the cells were washed 3 times for 15 minutes with 0.1 % Triton X-100 in PBS and stained with 0.1 mg/mL diaminophenylindole (DAPI) (Sigma). Olympus BX51 fluorescent microscope with appropriate filters and Cytovision software (Applied Imaging Corporation) was used for taking images. Three independent experiments were performed and the number of Ki67 positive cells was expressed as a percentage of total number of analyzed cells per treatment.

After incubation with mouse anti-tubulin antibody, the cells were washed 3 times for 15 minutes with 0.1 % Triton X-100 in PBS. Afterwards, the cells were incubated with biotinylated anti-mouse IgG antibody (Vector Laboratories, diluted 1:250 in 1 % BSA/0.1 % Triton X100 in PBS) for 1 hour at RT. After washing 3 times for 15 minutes with 0.1 % Triton X-100 in PBS, cells were incubated for 1h at RT with DyLight 594 Streptavidin antibody diluted 1:500 in PBS (Vector Laboratories). Later, cells were washing 3 times for 15 minutes with 0.1 % Triton X-100 in PBS and nuclei were stained with 0.1 mg/mL DAPI. Leica TCS SP8 confocal microscope applying Leica Microsystems LAS AF-TCS SP8 software (Leica Microsystems) was used for taking images.

### Chemical profiling

The phenolic profile of the extract was determined by LC-DAD-ESI/MSn (Dionex Ultimate 3000 UPLC, Thermo Scientific, San Jose, CA, USA). Compounds were separated and identified as previously described by Bessada et al. (2016[3]). A double online detection was performed using a DAD (280, 330 and 370 nm as preferred wavelengths) and a mass spectrometer (MS). The MS detection was performed in negative mode using a Linear Ion Trap LTQ XL mass spectrometer (Thermo Finnigan, San Jose, CA, USA) equipped with an ESI source. Phenolic compounds were identified based on their chromatographic behavior and UV-vis and mass spectra by comparison with standard compounds, when available, and data reported in the literature giving a tentative identification. Data acquisition was carried out with a Xcalibur® data system (Thermo Finnigan, San Jose, CA, USA). For quantitative analysis, a calibration curve for each available phenolic standard was constructed based on the UV-vis signal. For the identified phenolic compounds for which a commercial standard was not available, the quantification was performed through the calibration curve of the most similar available standard. The results were expressed as mg/g of the extract.

## Results and Discussion

### Enzyme inhibitory capacity of P. fruticosa

Extract of *P. fruticosa* has exhibited anticholinesterase activity towards both AChE (3.45 mgGALAE/g extract) and BChE (3.34 mgGALAE/g extract) (Table 1(Tab. 1)). A previous study of *Phlomis kurdica* essential oil (250 µg/mL) indicated that it could moderately inhibit both AChE and BChE (Karadağ et al., 2020[12]). On the other hand essential oils of *P. armeniaca*, *P. nissolii*, and *P. pungens var. pungens* were active against AChE with 0.667, 1.090 and 1.233 mg GALAE/g oil, respectively and to a lesser extent against BChE with 1.881, 3.032 and 2.521 mg GALAE/g oil, respectively (Sarikurkcu et al., 2016[26]). Different extracts of *P. armeniaca* have been able to inhibit AChE (0.534- 2.065 mg GALAEs/g extract) and BChE (1.436- 4.579 mg GALAEs/g extract) (Sarikurkcu et al., 2015[28]) as well as extract of *P. nissolii* (AChE, 0.471- 2.075 mg GALAEs/g extract, BChE 2.112- 3.247 mg GALAEs/g extract) and *P. pungens var. pungens* (AChE 0.483- 2.209 mg GALAEs/g extract, BChE 2.017-5.681 mg GALAEs/g extract) (Sarikurkcu et al., 2014[27]).

The extract exhibited significant tyrosinase inhibitory activity (133.16 mgKAE/g extract). Earlier studies highlighted the ability of different *Phlomis* species to interfere with the activity of tyrosinase: *P. caucasica* methanolic extract (IC_50_ 1.316 mg/mL, (Sarkhail et al., 2017[29])); *P. armeniaca* essential oil (66.723 mg KAE/g oil, (Sarikurkcu et al., 2016[26])); *P. armeniaca* extracts (9.88- 15.97 mg KAEs/g extract, (Sarikurkcu et al., 2015[28])); *P. nissolii* essential oil (63.301 mg KAE/g oil, (Sarikurkcu et al., 2016[26])); *P. nissolii* extracts (1.996- 14.210 mg KAEs/g extract, (Sarikurkcu et al., 2014[27])); *P. pungens* var. *pungens* essential oil (86.303 mg KAE/g oil, (Sarikurkcu et al., 2016[26])) and *P. pungens var. pungens* extracts (7.841- 29.560 mg KAEs/g extract, (Sarikurkcu et al., 2014[27])). 

To the best of our knowledge this is the first study of enzyme (AChE, BChE and tyrosinase) inhibitory activity of *P. fruticosa* methanolic extract. It has mainly shown similar anticholinesterase activity compared to previously tested *Phlomis* species but higher tyrosinase inhibitory potential (Sarikurkcu et al., 2014[27], 2015[28], 2016[26]).

### Antioxidant capacity of P. fruticosa 

Antioxidant properties of the *P. fruticosa* methanolic extract were examined by six different assays and obtained results revealed that the extract has strong antioxidant properties (Table 2(Tab. 2)).

Radical scavenging potential in DPPH assay was 39.3 mg TE/g extract, while for ABTS was 54.62 mg TE/g extract. Antiradical activity of different *P. fruticosa* extracts (aqueous, hydroalcoholic and alcoholic) were shown previously by DPPH assay (Ferrante et al., 2019[10]).

The total antioxidant capacity determined by phosphomolybdenum method was 1.22 mmol TE/g extract. The reducing powers determined by CUPRAC and FRAP assays indicated activity of the extract in 123.44 mg TE/g extract and 71.17 mg TE/g extract, respectively. A previous study of *P. armeniaca* methanolic extract (Sarikurkcu et al., 2015[28]) has determined its activity in CUPRAC assay at 127.60 mg TEs/g extract and FRAP assay at 87.08 mg TEs/g extract which is slightly higher compared to the detected antioxidant capacity of the *P. fruticosa* methanolic extract (Table 2(Tab. 2)).

Metal chelating activity of the *P. fruticosa* extract was determined at 13.16 mg EDTAE/g extract. Literature data revealed that the methanolic extract of *P. armeniaca* has exhibited chelating effect at 20.40 mg EDTAEs/g extract (Sarikurkcu et al., 2015[28]), *P. nissolii* at 17.01 mg EDTAEs/g extract, while *P. pungens *var.* pungens* extract at 21.08 mg EDTAEs/g extract (Sarikurkcu et al., 2014[27]) suggesting the lower activity of the *P. fruticosa *extract.

### Anti-glioblastoma activity of P. fruticosa 

**Figure 1:**
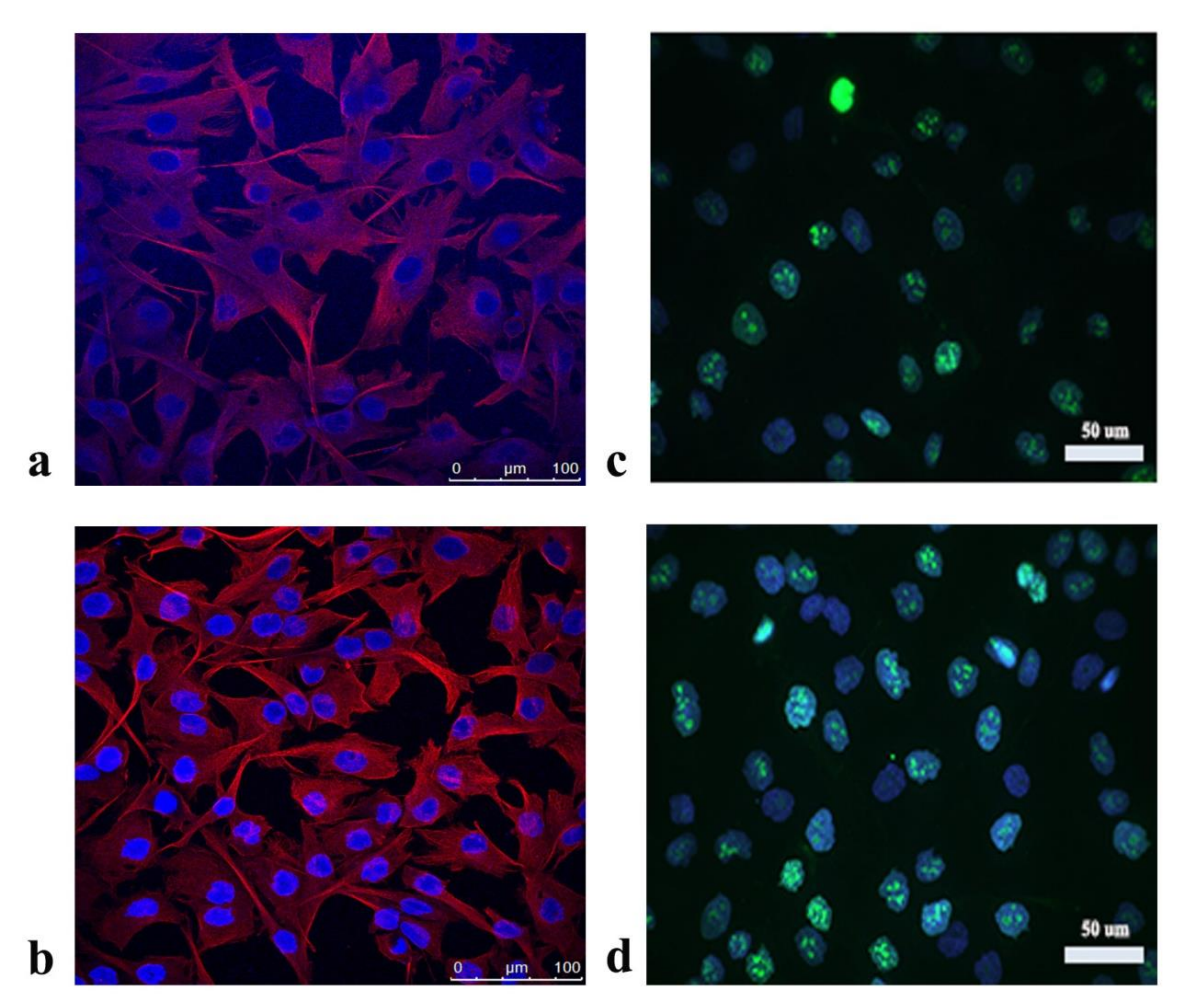
Morphology of A172 cells analyzed by fluorescence staining of cytoskeletal protein tubulin after treatment with a) *P. fruticosa* extract, b) DMSO - vehicle control; and the expression of Ki67 protein after application of c) *P. fruticosa* extract, d) DMSO - vehicle control

By Crystal violet assay we determined concentration of the methanolic extract required for 50 % inhibition of the A172 growth (IC_50_ 710.83±63.36 μg/mL). Application of the extract has no effect on cell morphology examined by staining with the cytoskeletal protein tubulin (Figure 1a, b(Fig. 1)), nor cell proliferation, as determined by analysis of the Ki67 protein expression (Figure 1c, d(Fig. 1)). A previous study (Stojković et al., 2020[33]) has determined weak to prominent cytotoxic potential of the *P. fruticosa* extract towards different cancer cells (MCF7, SiHa, HepG2) accompanied by lack of cytotoxicity towards human primary cells HGF-1 (IC_50_ > 800 μg/mL). These data indicated that the *P. fruticosa* extract has weak cytotoxic potential. 

**Figure 2:**
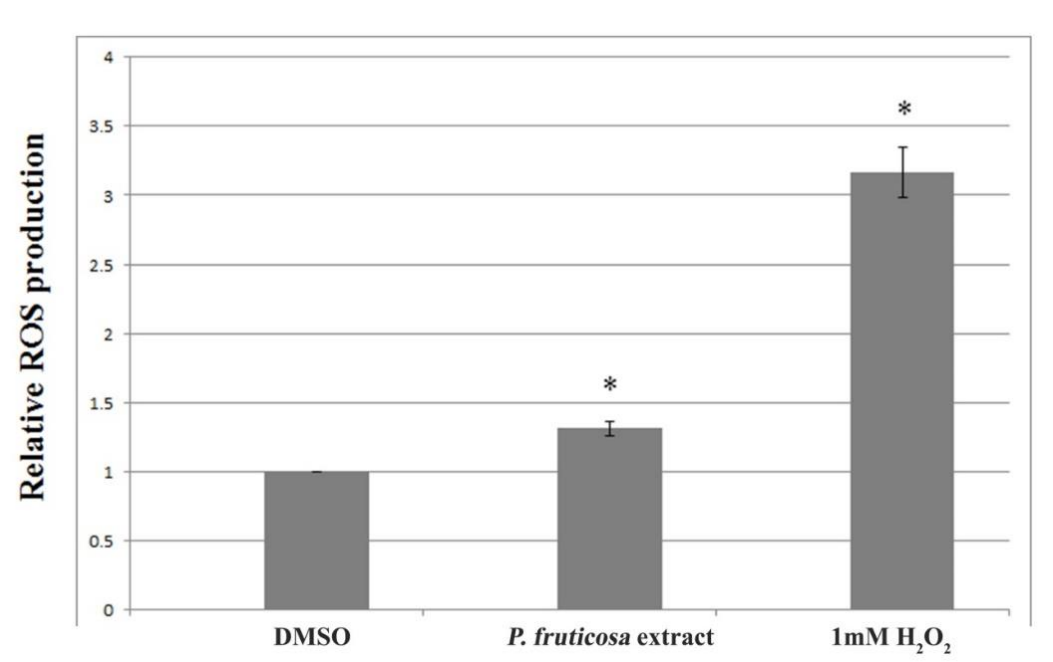
Effect of the *P. fruticosa* extract on ROS production in A172 cells. Relative ROS production was calculated compared to DMSO treated-A172 cells which were set as 1. *p < 0.05

Results obtained by dichlorofluorescein assay shown that A172 cells exposed to IC_50_ concentration of the *P. fruticosa* extract demonstrated an increase in the ROS production (Figure 2(Fig. 2)). These data suggest that the observed cytotoxic effect of the *P. fruticosa* extract on A172 cells might be, to some extent, based on the increase in ROS production leading to cancer cell death.

### Chemical constituents

Our previous investigation revealed detailed chemical profile of the *P. fruticosa* methanolic extract by using UHPLC-LTQ-Orbitrap/MS analysis and 44 different phenolic constituents were detected (Stojković et al., 2020[33]). The individual phenolic compound profiles of several *Phlomis* species have been previously studied and reported in the review article by Limem-Ben Amor et al. (2009[17]) that overviews and complies the phytochemical profile and some biological activities of the *Phlomis *species. Marin et al. (2007[18]) analyzed flavonoids in *P. fruticosa* from Montenegro, while Ersöz et al. (2002[9]) focused on the description of iridoid compounds and phenylpropanoid glycosides from *Phlomis grandiflora *var. *fimbrilligera *and *P. fruticosa*. In the present study, a detailed description of the phenolic compounds found in the methanolic extract of *P. fruticosa* was performed by HPLC-DAD/ESI-MSn, and the chromatographic data obtained regarding retention time, UV-Vis spectra, molecular ion, fragmentation pattern, as also the tentative identification and quantification (mg/g extract) are presented in Table 3(Tab. 3).

Twelve phenolic compounds were found including four phenolic acids (chlorogenic acid derivatives), five phenylpropanoid glycosides, and three flavonoids (*C*-glycosylated apigenin and *O*-glycosylated luteolin derivatives). Due to the sensitivity of Orbitrap technology more compounds were found in the *P. fruticosa* methanolic extract in our previous analysis (Stojković et al., 2020[33]), indicating their presence only in trace amounts, since we were not able to detect all of them in the current HPLC-DAD/ESI-MSn analysis.

Regarding phenolic acids group, peak **4** ([M-H]^-^ at *m/z* 353) was assigned as 5-*O*-caffeoylquinic acid (chlorogenic acid) by comparison of its UV spectra and retention time with the available standard compound. For the tentative identification of peaks **1 **and **2**, that presented the same pseudomolecular ion [M-H]^-^ at *m/z* 353 and similar fragmentation pattern as peak **4**, the hierarchal key developed by Clifford et al. (2003[6]) for the identification of chlorogenic acid derivatives was used, and peaks 1 and 2 has been assigned as *cis* and *trans* form of 3-*O*-caffeoylquinic acid. Chlorogenic acid derivatives, especially caffeoylquinic acids were previously described in several species of *Phlomis* (Limem-Ben Amor et al., 2009[17]). Finally, peak **5** ([M-H]^-^ at *m/z* 337), presented a similar fragmentation pattern as peaks **1**, **2**, and **4**, but the linkage to the quinic moiety is with a *p*-coumaroyl acid group, has been tentative identified as 5-*O*-*p*-coumarouylquinic acid, as previously described by Clifford et al. (2006[7]).

The phenylpropanoid glycoside group was the most numerically abundant compound, gathering peaks **7** ([M-H]^-^ at *m/z* 755), **8** ([M-H]^ -^ at *m/z *623), and **10** ([M-H]^ -^ at *m/z *769). These compounds have been previously identified in *Phlomis grandiflora *var. *fimbrilligera *and *P. fruticosa* by Ersöz et al. (2002[9]), and following this description they are tentatively identified as forsythoside B, verbascoside isómer 1, and alyssonoside, respectively. Peak **9** presented the same chromatographic behavior as peak **8** (verbascoside), with a pseudomolecular ion [M-H]^ -^ at *m/z *623 and the same MS^2^ fragments at *m/z* 461 and 315. Kirmizibekmez et al. (2004[13]) also found two peaks with a close retention time and with the same chromatographic behavior in *Phlomis brunneogaleata* and based on this peak **9 **was tentatively identified as isoverbascoside. Peak **11**, also presenting the same chromatographic response as peak **8** and **9**, was tentatively identified as an isomer of verbascoside. This type of identification has been previously described by other authors (Li et al., 2014[14]). 

Finally, for the group of flavonoids two *C*-glycosylated apigenin derivatives were found in the studied sample, peaks **3** ([M-H]^-^ at *m/z* 593) and **6** ([M-H]^-^ at *m/z* 563). Peak **3** was tentatively assigned as apigenin-6,8-*C*-dihexoside, presenting a base peak at *m/z* 473 [(M−H)−120]^−^, *m/z* 353 [(M−H) − (120 + 120)]^−^ and *m/z* 383 [(M−H) − (90 + 120)]^−^, indicating the presence of apigenin aglycone linked to two hexose moieties; this fragmentation pattern is typical of di-*C*-glycosyl flavones (Tahir et al., 2012[35]). Peak **6**, was tentatively assigned as apigenin-*C*-hexoside-*O*-pentoside, also presenting a typical fragmentation pattern of *C*-glycosyl derivatives; however the type of sugar and linkage is different than that of peak **3**. This differentiation followed the information previously described by Tahir et al. (2012[35]) and Ferreres et al. (2018[11]). The only one luteolin was found in the sample (Peak **12,** [M-H]^-^ at *m/z* 461) tentatively assigned as luteolin-*O*-glucuronide, revealing an unique MS^2^ fragment at *m/z *285 (luteolin aglycone), corresponding to the loss of the glucuronyl moiety (loss of -176 u). 

Regarding the quantification of the detected phenolic compounds, although phenylpropanoid glycosides were the most numerous, that do not correspond to a higher concentration of these compounds. In fact, phenolic acids group presented the highest concentration (6.06±0.02 mg/g extract), mainly due to the presence of peak **1** (*cis* 3-*O*-caffeoylquinic acid, 4.2±0.1 mg/g extract).

See also the Supplementary data.

## Conclusions

The presented study highlighted the *P. fruticosa* methanolic extract as a promising bio-therapeutic. The extract reduces activities of enzymes associated with neurodegenerative diseases exhibiting *in vitro* antineurodegenerative activity. The extract also displayed antioxidant activity and induced ROS production in glioblastoma cell line. Chemical analysis revealed wide pallet of polyphenols, with *cis-*3-*O*-caffeoylquinic acid as the most abundant representative. Biological properties of the *P. fruticosa* extract presented in this study could be at least partly attributed to the presence of *cis *3-*O*-caffeoylquinic acid as the most dominant compound. Caffeoylquinic acids have been found in many plant species which are components in the daily diet and exhibit a wide spectrum of biological activities, including antioxidant, immunomodulatory, antihypertensive, analgesic, anti-inflammatory, hepato- and neuroprotective, anti-hyperglycemic, anticancer, antiviral and antimicrobial activities (Skała et al., 2020[30]). Obtained *in vitro* data provides solid support for future prospective *in vivo* studies in order to reveal full capacity of this natural polyphenol-rich bioactive agent.

## Declaration

### Conflict of interest 

The authors declare no conflict of interest.

### Acknowledgments

This work has been supported by the Ministry of Education, Science and Technological Development of the Republic of Serbia (451-03-9/2021-14/200007 and 451-03-68/2022-14/200042). The authors are grateful to the Foundation for Science and Technology (FCT, Portugal) and FEDER under Program PT2020 for financial support to CIMO (UIDB/00690/2020); national funding by FCT, P.I., through the institutional scientific employment program-contract for M.I. Dias and L. Barros; to FEDER-Interreg España-Portugal program for financial support through the project 0377_Iberphenol_6_E and TRANSCoLAB 0612_TRANS_CO_LAB_2_P.

## Supplementary Material

Supplementary data

## Figures and Tables

**Table 1:**
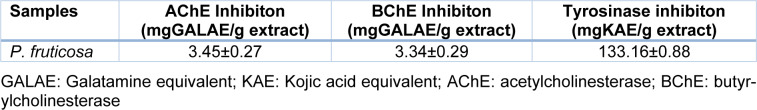
Enzyme inhibitory properties of *Phlomis fruticosa* methanolic extract (mean±SD)

**Table 2:**
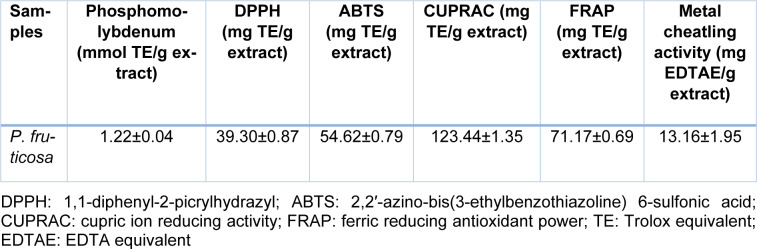
Antioxidant properties of *Phlomis fruticosa* methanolic extract (mean±SD)

**Table 3:**
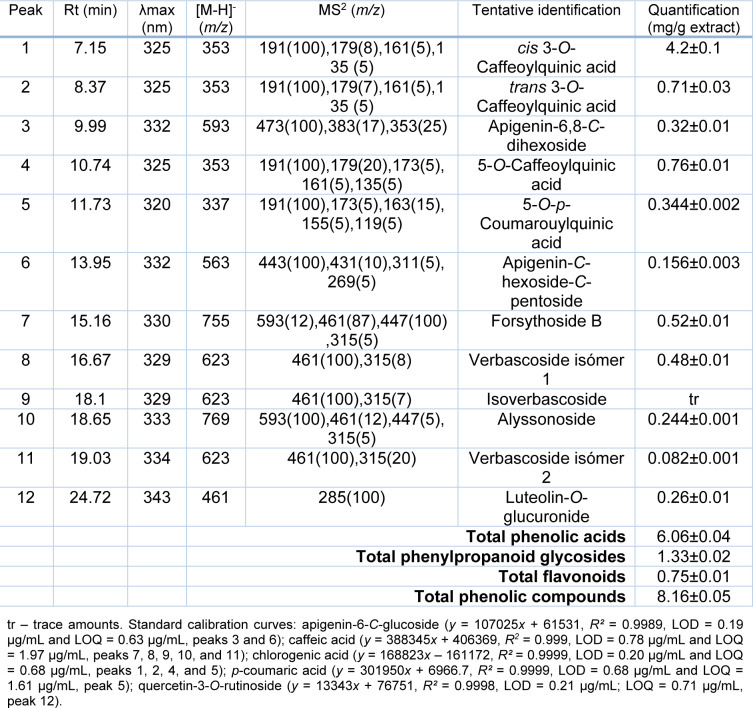
Retention time (Rt), wavelengths of maximum absorption (λ_max_), mass spectral data, tentative identification, and quantification (mg/g extract) of the phenolic compounds present in the extracts of *P. fruticosa.*
